# Effect of early vitamin D supplementation on the incidence of preeclampsia in primigravid women: a randomised clinical trial in Eastern Democratic Republic of the Congo

**DOI:** 10.1186/s12884-024-06277-6

**Published:** 2024-02-03

**Authors:** Richard Kabuseba Kabuyanga, Pierrot Lundimu Tugirimana, Balungwe Sifa, Mwanga Balezi, Michel Ekanga Dikete, Prudence Ndeba Mitangala, Jean Pierre Moyene Elongi, Xavier Kalume Kinenkinda, Jean-Baptiste Sakatolo Zambeze Kakoma

**Affiliations:** 1 Department of Gynecology-Obstetrics, University of Goma, Goma, Democratic Republic of the Congo; 2 Department of Laboratory & Basic Sciences, University of Goma, Goma, Democratic Republic of the Congo; 3 Department of Gynecology-Obstetrics, Panzi Hospital, UEA, Bukavu, Democratic Republic of the Congo; 4 Mwesso General Referral Hospital, Masisi, Democratic Republic of the Congo; 5 Department of Gynecology-Obstetrics, Free University of Brussels, University Clinic of Brussels, Erasmus Hospital, Brussels, Belgium; 6 Public Health Department, Université Officielle de Ruwenzori, Butembo, Democratic Republic of the Congo; 7 Department of Gynecology-Obstetrics, General Hospital of Kinshasa, Kinshasa, Democratic Republic of the Congo; 8 Department of Gynecology-Obstetrics, University of Lubumbashi, University Clinics of Lubumbashi, Lubumbashi, Democratic Republic of the Congo; 9 Department of Gynecology-Obstetrics and School of Public Health, University of Lubumbashi, University Clinics of Lubumbashi, Lubumbashi, Democratic Republic of the Congo

**Keywords:** Preeclampsia, Prevention, Vitamin D, Supplementation

## Abstract

**Background:**

Previous studies have reported the association between maternal vitamin D deficiency and preeclampsia. However, the efficacy of vitamin D supplementation in reducing the occurrence of preeclampsia remains unclear. The objective of this study was to evaluate the effect of cholecalciferol supplementation on the incidence of preeclampsia in primigravid women and its related maternal and foetal outcomes.

**Methods:**

A single-blinded clinical trial was conducted in fourteen antenatal care health facilities in the North (Goma, Mwesso, Nyiragongo) and South Kivu (Bukavu-Panzi) provinces of the Democratic Republic of Congo from March 1, 2020, to June 30, 2021. A total of 1300 primigravid women not exceeding 16 weeks of gestation were randomised with a 1:1 ratio to either the supplemented (A) or control (B) group. Each pregnant woman (A) presenting for antenatal care received a single monthly dose of cholecalciferol (60,000 IU) orally for 6 months. The control group received no vitamin D supplementation or placebo. Serum 25(OH)D was measured at recruitment and at 34 weeks of gestation. Outcomes were assessed monthly until delivery.

**Results:**

The median maternal age was 21 years (14–40), while the median gestational age was 15 weeks (5.4–29.0). A significant reduction in the risk of preeclampsia [RR = 0.36 (0.19–0.69); *p* = 0.001] and preterm delivery [RR = 0.5 (0.32–0.78); *p* = 0.002] was observed in the intervention group. An RR of 0.43 [(0.27–0.67); *p* < 0.001] was found for low birth weight. The RR for caesarean section was 0.63 [(0.52–0.75); *p* < 0.001]. The APGAR score at the 5th minute (*p* = 0.021) and the size of the newborn were significantly higher in the supplemented group (*p* = 0.005).

**Conclusion:**

A single monthly dose (60,000 IU) of vitamin D supplementation, started in earlypregnancy, significantly reduced the incidence of preeclampsia and its maternal and foetal complications.

**Trial registration:**

ISRCTN Register with ISRCTN46539495 on 17 November 2020.

## Background

Hypertensive disorders of pregnancy, including preeclampsia (PE), affect approximately 10% of pregnant women worldwide [1], and it is estimated that preeclampsia affects 4.6% of pregnant women [2]. Annually, approximately 70,000 women and 500,000 newborns die as a result of preeclampsia and other hypertensive disorders of pregnancy [3–5]. Preterm delivery and low birth weight are dreaded consequences related to this condition [6]. The prevalence of preeclampsia (PE) at the Provincial Hospital of North Kivu in Eastern Democratic Republic of Congo was 3.01% in 2019 [7].

Although the main cause of PE remains unknown, progress has been made in the understanding of the pathophysiological mechanisms leading to the disease and in the identification of its risk factors [8]. Early detection of PE in the laboratory is just as challenging as treatment. A test based on the assay of endothelial progenitor cells (EPCs) and natural killer cells in peripheral blood has produced promising results [9]. However, recently (2022), another early detection test for PE (Congo Red Dot Paper Test) with high predictive value (high specificity and sensitivity) and highly suitable for environments with low resources has been developed [10]. Preventive and curative treatments for preeclampsia have long been a challenge. Early detection of PE coupled with the recognised pharmacological properties of vitamin D [11] is a first-line strategy to reduce the adverse maternal and foetal impacts of PE, which is a multifactorial disease. The results from observational studies have shown that low vitamin D levels are associated with preeclampsia [12, 13]. Similarly, studies have revealed an association between vitamin D deficiency and low birth weight, preterm delivery and gestational hypertension [14]. Moreover, vitamin D deficiency has been linked to the early onset of severe PE [15]. In 2014, globally, approximately 88.1% of the world's population was reported to have 25(OH)D (Calcidiol or Calcifediol) levels below 30 ng/mL [16], and this deficiency also occurs in pregnant women [17].

As there is no available curative treatment other than delivery, an intervention with the ability to prevent PE would have a substantial impact on maternal and infant health global [18]. Recent findings suggest that vitamin D has properties that can alleviate the pathogenic mechanisms that generate PE [19]. Few studies on the estimated need, efficacy, safety and benefits of maternal–foetal vitamin D supplementation have been conducted with the aim of implementing a prenatal screening and supplementation programme. Hollis et al. as well as those before it have clearly demonstrated vitamin D to be safe at up to 4000 IU/d for pregnant women [20–23]. Therefore, vitamin D supplementation could be recommended to reduce the incidence of PE. Margaretha et al. found that supplementation with 10–15 µg/d vitamin D was associated with a 27% reduction in the risk of preeclampsia in a series of nulliparous women [24]. In the Central and East African region, there are no data on vitamin D and supplementation in pregnant women. However, sufficient evidence is still lacking [25].

The aim of this study was to evaluate the effect of vitamin D supplementation administered in early pregnancy on the incidence of PE in primigravidae as well as to evaluate adverse maternal-foetal-neonatal outcomes usually observed in patients with preeclampsia.

## Methods/design

### Study design and setting

This was a multicentre, single-blind, randomised controlled trial. The study was approved by the ethical committee of the University of Lubumbashi (N° UNILU/CEM/125/2019 of February 8, 2019) and by both provincial health divisions of North-Kivu and South-Kivu (N° 251/281/DPS-NK/2019 of 19/06/2019 and N° 008/CD/DPS-SK/2020). The ANC centres where this clinical trial is being carried out are under the supervision of the provincial health authority which, after analysing the protocol, granted official authorisation. However, each participant was asked to sign an individual consent form. The study was financed exclusively by its authors.

### Study population

Pregnant women were recruited from 14 hospitals and health centres offering antenatal care (ANC) in the cities of Goma (North Kivu) and Bukavu (South Kivu) in Eastern Democratic Republic of Congo. These included the Provincial Hospital of North Kivu, La Charité Maternelle Hospital, Virunga Hospital, Kyeshero Hospital, Heal Africa Hospital, Mwesso Hospital, Panzi Hospital, Kanyaruchinya Health Center, Majengo Health Center, Murara Health Center, Kahembe Health Center, Mabanga Health Center, Kiziba Health Center, and Hope Wellness Center.

### Inclusion criteria

Participants were enrolled from March 1st, 2020, to June 30th, 2021. They were screened for eligibility when they presented to the health facility for ANC visits during regular working hours and were offered enrolment if they met the following inclusion criteria: were a primigravida woman carrying a singleton pregnancy not exceeding 16 GW, estimated based on the first day of the last menstrual period; and were residing at a fixed address and planning regular follow-up and delivery at the same health facility. Telephone contact for pregnancy follow-up was maintained by the principal investigator based on the contact details provided by the pregnant woman or her spouse.

Vitamin D supplementation, no later than 16 GW, is justified by the aim of possibly obtaining its effects on the deficient trophoblastic invasion process incriminated in the pathogenesis of PE.

### Exclusion criteria

The exclusion criteria were preeclampsia diagnosed at the time of inclusion, multiple pregnancy, pathologies (hypertension, tuberculosis, sickle cell disease, maternal malnutrition, etc.) with an impact on foetal growth, declared supplementation with pharmacological preparations containing vitamin D and/or calcium, a history of hepato-renal diseases or diabetes mellitus and nonconsent to participate in the study.

### Sample size

The prevalence of preeclampsia is estimated at 8.5% [26]. We projected a 50% reduction in the prevalence of preeclampsia by providing vitamin D supplementation for pregnant women. With a power of 80% and an alpha error of 5%, a minimum sample size of 241 pregnant women was needed, i.e., 120.5 pregnant women per group (STATA 14.2/MP).

*n* = the number of subjects required in each group.

${I}_{ne}$= the expected incidence of the phenomenon among the exposed individuals.

Z_α_ = the value of Z for first-case risk (α = 5%, Z_α_ = 1.96).

Z_2ß =_ the value of Z for a power of 1-ß (for a power of 80%, ß = 20% and Z2ß = 0.84).

RR = the minimum relative risk for the study to be of public health significance is 2.

p = the average incidence of disease in both groups.

I_ne_ = 0.085 (8.5%), *p* $$=\frac{0.085\left(1+2\right)}{2}$$=0.128.

n $$\ge \frac{{\left[\left[1.96\sqrt{2*0.128 (1-0.128}\right]+0.84\sqrt{0.085+\left(0.085\right)*(2)-{{0.085}^{2 }-{0.085}^{2 }2}^{2}]}\right]}^{2}}{[{{0.085}(1-2)]}^{2}}$$=240.8

In each group, n was ≥ 241. Considering expected losses (loss to follow-up), this calculated minimum sample size (*n* = 241) was increased by 10% (241 + 25 = 266).

### Statistical analysis

Data were recorded into a Microsoft Excel (Microsoft Corporation, Redmond, WA) 2019 file and then exported to SPSS (version 23; SPSS Inc., Chicago, USA) for analysis. The normality of the distribution was tested prior to the following calculations and tests: percentages, means with standard deviations, medians and interquartile ranges, Pearson chi-square tests, and relative risks (RRs) with 95% confidence intervals. The *p* values less than 0.05 will be considered statistically significant.

An intention-to-treat approach was employed in the analysis of the primary outcome, including all data irrespective of a participant’s adherence or duration of supplementation.

### Randomization and blinding

Patients were assigned in a 1:1 ratio to the vitamin D supplementation group or the control group. One staff member with no role in the study managed the randomisation, and another managed the administration of pharmaceuticals to the pregnant women, including vitamin D. The randomisation list was obtained using a computer-generated code with block sizes of 4. Numbers were randomly generated in Excel office 2010 software and randomly assigned exclusively to primigravidae up to 16 weeks of gestation, allowing for their allocation into the two groups in an alternative way. Sociodemographic information, clinical data, and baseline blood and urine samples were obtained at the initial visit following subject consent. No financial contribution was obtained from the participants. Nor were they paid on this occasion. It should also be noted that the study was not advertised.

### Study interventions, baseline evaluation and follow-up

The vitamin D group received a single monthly oral dose of 60,000 IU vitamin D (Vitossamin® D3, Alisons, Olive health care, Toronto, Canada; Daman, India). Each woman in the supplemented group received a total of six doses over a period of six months. Supplementation started at 16 weeks of gestation at the latest and at 12 weeks of gestation at the earliest. The cholecalciferol capsule was administered by the pharmacist during antenatal care visits. The intervention was single-blinded; only the assessors were unaware of each participant’s randomised group.

All pregnant women were followed and monitored on a monthly basis until delivery. The second blood sample was taken at 34 weeks of gestation.

Pregnant women in the control group received no placebo. We also excluded any primigravid women that had received vitamin D supplementation.

The study personnel were trained in advance for data collection and biological sample processing outcome assessment.

The primary outcome for this trial was the incidence of preeclampsia, and the secondary outcomes were preterm delivery, birth weight and height, mode of delivery, and APGAR score. The viability index (APGAR) was assessed at 1, 5 and 10 min after delivery. The umbilical cord and placenta are the foetal annex for which information has been collected for later publication.

Preeclampsia was defined as hypertension (SBP ≥ 140 and/or DBP ≥ 90 mm Hg) associated with proteinuria measured by dipstick (≥ 2 crosses) at 20 GW [27]. Preterm delivery was defined as delivery occurring before 37 completed weeks of gestation. Low birth weight was diagnosed in any newborn with a weight below the 10th percentile for gestational age or less than 2500 g [28].

### Blood collection, processing, and analysis

Two blood samples were collected from each primigravida for the determination of 25(OH)D and calcium levels. The first sample was taken at inclusion and the second was taken at the 34th week of gestation. Antecubital venous blood samples were drawn into 4 mL tubes that contained no anticoagulant. Blood was allowed to clot at room temperature for 30 min and then placed on ice for transport to the hospital for centrifugation. The obtained serum was aliquoted and frozen at -80 °C for later analyses at the Provincial Hospital of North Kivu. The measurements of serum 25(OH)D levels were performed using ichroma™ II (Boditech Med Inc., Chuncheon, Republic of Korea), an automated point-of-care fluorescence immunoassay (FIA) for the quantitative determination of total 25(OH)D2/D3 levels in human serum or plasma. The consensus for the clinical classification of vitamin D levels was applied: deficiency (< 8 ng/mL), insufficiency (8 to 29.9 ng/mL), and sufficiency (30 ng/mL or more). The measurements of calcium were performed using an automated electrolyte analyser, GE300 (Genrui, Shenzhen, China). Quality control checks were conducted using quality materials provided by the manufacturers.

## Results

**Fig. 1:**
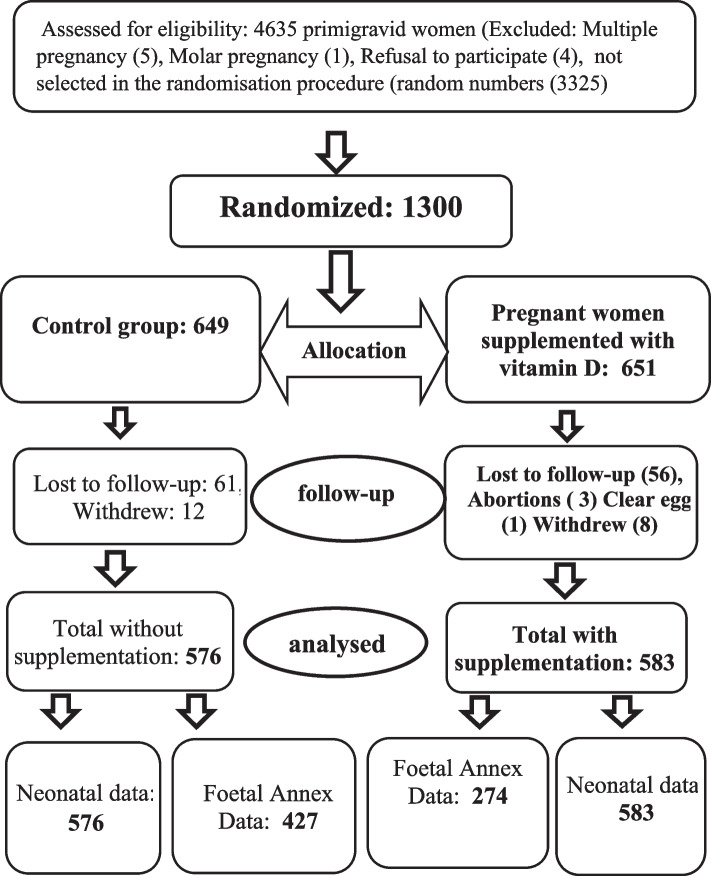
Flow chart of the subjects throughout the study

Among the 1300 participants recruited and randomly assigned to either the intervention or the control group, 156 were lost to follow-up: 73 in the control group and 68 in the vitamin D supplementation group (Fig. 1). At the end of the study, we included 1159 pregnant women: 576 who were nonsupplemented and 583 who received monthly vitamin D supplementation. The placenta and umbilical cord were the two foetal annexes selected for this study and will be the subject of a future publication.

Participant characteristics and 25(OH)D and calcium levels at baseline were similar in both arms (Table 1).

**Table 1:** Maternal Sociodemographic and Clinical Characteristics at Study Enrolment

Parameters	Global (*n* = 1159)	Intervention group (583)	Control group (576)	*p*
**Maternal age,** years				**0.81**
*Median (Min–Max****)***	21 (14–40)	21 (15–38)	21 (14–40)	
**Education level**^**a**^	**% (*n* = 1157)**	**(*n* = 582)**	**(*n* = 575)**	**0.18**
*Low*	53.5 (619)	51.4 (299)	55.7 (320)	
*Mean*	27.8 (322)	30.2 (176)	25.3 (146)	
*High*	18.7 (216)	18.4 (107)	19.0 (109)	
**GA at inclusion** (weeks) Median (Min, Max)	12.6 (5.4–16.1)	12.6 (5.4–16.0)	13.0 (6.0–16.1)	**0.809**
**History of alcohol use**	9.5 (110)	10.2 (59)	8.9 (51)	**0.46**
**Cigarette smoking history**	0.4 (5)	0.3 (2)	0.5 (3)	**0.69**
**SBP mm Hg**		107.05 ± 79	106.9 ± 97	**0.79**
**DBP mm Hg**		67.32 ± 10	67.15 ± 79	**0.73**
**Measurement of 25(OH)D at inclusion**		34.20 ± 11.9	35.61 ± 12.7	**0.051**
**Ionised calcium at inclusion**		1.16 ± 0.16	1.19 ± 0.16	**0.005**

^a^Education: *(low: none up to incomplete secondary level; mean: complete secondary and vocational training; high: complete or incomplete university*

This was a broadly homogenous population.

The incidence of preeclampsia in the supplemented group (2.1%) was significantly lower at the end of the trial than that in the nonsupplemented group (5.7%). The protective effect of vitamin D was reflected in the fact that the risk of developing preeclampsia in the nonsupplemented group was approximately three times greater than that in the supplemented group in our series (relative risk (RR) = 2.8). Thus, the observed protective effect is supported by the finding that the nonsupplemented group was the major contributor of preeclampsia patients in this cohort, accounting for 73.3% of cases (33/45). In addition, the difference in mean vitamin D levels at 34 weeks gestation between the two groups was highly significant (*p* < 0.001), although there was no indication of any fluctuation in the levels before the second sampling. The caesarean delivery rate was significantly high in the study population (29.8%), with a high contribution from the control group (61.2%). In the same cohort, vitamin D supplementation significantly reduced the risk of caesarean delivery by 37%. The risks of preterm delivery and induction of labour were two and forty times higher, respectively, in the nonsupplemented group than in the supplemented group, with the difference being statistically significant (Table 2).

**Table 2:** Vitamin D, ionised calcium, type of delivery and gestational outcomes

Parameters	Nonsupplemented % (n)	Supplemented % (n)	RR (CI 95%)	*p*
**Mode of delivery**				
*Spontaneous vaginal delivery*	57.6 (332)	76.8 (448)		
*Caesarean section*	36.6 (211)	23.0 (134)	0.63 [0.52—0.75]	< 0.001
*Induced labour (Misoprostol)*	5.7 (33)	0.2 (1)	0.025 [0.003–0.18]	< 0.001
**Preeclampsia**	5.7 (33)	2.1 (12)	0.36 [0.19—0.69]	0.001
**Preterm birth**	9.5 (55)	4.8 (28)	0.50 [0.32–0.78]	0.002
**Measurement of 25(OH)D**^**a**^	41.47 ± 20.3	37.25 ± 9.4	**-**	0.000
**Ionised calcium**^**a**^	1.17 ± 0.19	1.17 ± 0.15	**-**	0.532

^**a**^**at the end of the supplementation**

**Fig. 2:**
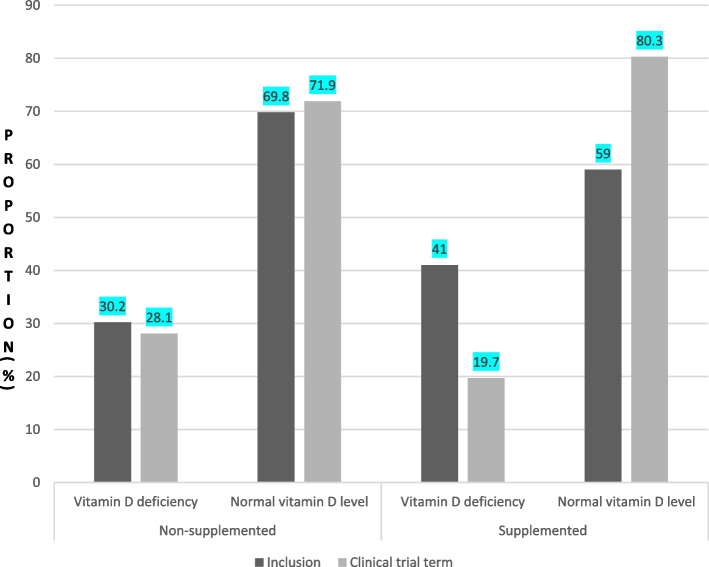
Distribution of pregnant women by vitamin D levels and allocations at inclusion and at the end of the RCT

In the supplemented group, the proportion of participants with vitamin D deficiency fell from 239 (41%) at the start of the study to 115 (19.7%) at the end. In contrast, the proportion in the control group did not fall markedly at the start 174 (30.2%) or end of the study 162 (28.1%) (Fig. 2). The mean time interval between inclusion and the first measurement was 18 weeks.

The incidence of complications typically seen in women with preeclampsia was less frequent in the supplemented group than in the nonsupplemented group. The likelihood of preterm delivery was significantly greater in the nonsupplemented group (9.5%) than in the cohort as a whole (Table 2). The control group accounted for 73.3% of the cases of preeclampsia. When considering women with preeclampsia, the proportion with a vitamin D level below 40 ng/mL was 60.6% in the supplemented group and 58.3% in the nonsupplemented group. The earliest delivery in the cohort occurred after 24 weeks of gestation (WG), and the latest occurred after 43 WG. For gestation week at delivery, the mean ± SD was slightly but significantly longer in the supplemented group (38.91 ± 1.4 WG) than in the control group (38.62 ± 2.1 WG; p [95% CI] = *P* < *0.001* [-0.49 –—0.08] (Table 2).

Neonatal outcomes were generally good, with an overall proportion of 7.4% (86/1159) of newborns with low birth weight (10.4% in the nonintervention group and 4.5% in the supplemented group), and the difference was highly significant. The protection against the risk of low birth weight provided by vitamin D supplementation was thus more than twice as high in the supplemented group compared with the nonsupplemented group (RR = 2.3). However, the mean birth weight was not significantly different between the two groups (3069.48 ± 419.44 g vs. 3039.06 ± 504.7 g; *p* = 0.265 and 95% CI (-83.9–23.1)), with birth weights ranging from 1200 to 5000 g for the supplemented group and 1000 to 4800 g for the nonsupplemented group. The newborns in the supplemented group had significantly greater adaptability to extrauterine life compared to those in the control group (*p* = 0.021). The difference was also significantly greater for the statural growth index (*p* = 0.005) (Table 3).

**Table 3:** Vitamin D supplementation and neonatal outcomes

Parameters	Nonsupplemented % (n)	Supplemented % (n)	RR (CI 95%)	*p*
**Sex**				
*Male*	50.7 (292)	50.5 (294)	**-**	
*Female*	49.3 (284)	49.5 (289)	**-**	
**Neonatal birth weight**				
*Low birth weight,*	10.4 (60)	4.5 (26)	0.43 (0.27–0.67)	< 0.001
*Normal weight*	87.2 (502)	92.6 (540)		
*Macrosomia*	2.4 (14)	2.9 (17)	1.20 (0.60–2.41)	0.72
**Birth length***, cm (Mean* ± *SD)*	48.5 (2.7)	48.9 (2.3)		0.005
**Head circumference,** *cm (Mean* ± *SD)*	34.6 (1.8)	34.7 (1.4)		0.14
**Apgar score at 1 min** *(Mean* ± *SD)*	8.4 (1.5)	8.6 (1.3)		0.011
**Apgar score at 5 min**	9.2 (1.5)	9.4 (1.2)		0.021
**Apgar score at 10 min**	9.7 (1.2)	9.8 (1.1)		0.024

## Discussion

Although the relationship between vitamin D and obstetric diseases has been studied intensively worldwide, data from the African continent remain scarce. The present study of pregnant women is the first of its kind to have been conducted in the Democratic Republic of Congo. Although observational studies have provided important information on the relationship between preeclampsia and maternal vitamin D levels, intervention (supplementation) trials are likely to provide reliable guidance for professional practice. However, the published trials based on vitamin D supplementation in pregnant women vary markedly with regard to several methodological aspects: the study population (parity, the presence or absence of established vitamin D deficiency), the gestational age at inclusion, the formulation of the vitamin D administered (injectable vs. oral), the supplementation duration, frequency and dose, vitamin D supplementation alone or combined with other substances (antioxidants, minerals, etc.), and the essay method.

Our decision to study primigravidae was dictated by the known importance of a first-ever pregnancy as a risk factor for preeclampsia and its unambiguous definition, which reduced confusion during recruitment of the study population. Furthermore, our administration of vitamin D at the end of the first trimester of pregnancy was in line with the vitamin's expected effect on the pathogenesis of preeclampsia.

Below, we shall solely discuss literature data on supplementation with vitamin D alone, rather than in combination with other substances.

With regard to the study's primary outcome, we found that vitamin D supplementation protected against preeclampsia. This finding is in line with the results of a study in Norway that revealed a 27% reduction in the risk of preeclampsia (odds ratio (OR) [95% CI] = 0.73 [0.58–0.92]) in pregnant women taking 10–15 µg/d vitamin D relative to those not taking it. However, the researchers did not observe an association between the risk of preeclampsia and the amount of vitamin D obtained from the diet [24].

A protective relationship was also observed in Sasan et al.'s randomised controlled trial (RCT) of 142 pregnant women with a history of preeclampsia conducted in Iran. The pregnant women included in the intervention arm received 50,000 IU vitamin D twice a month. The recurrence of preeclampsia was significantly more frequent in the nonsupplemented group (30.6%) than in the supplemented group (15.7%; *p* = 0.036; RR [95% CI] = 1.94 [1.02–3.71]) [29]. The agreement of our results might be due to the known benefits of a substantial dose of vitamin D administered sufficiently early, i.e., immunomodulatory actions and effects on the transcription and function of genes (e.g., *VEGF*) that are associated with normal placental implantation, invasion, and angiogenesis [29].

In contrast, Naghshineh et al.'s RCT of 138 primigravidae (600 IU vitamin D daily from 16 WG until delivery in the intervention arm) did not show a significant difference (*p* = 0.17) in the incidence of preeclampsia – although 78% of cases of preeclampsia occurred in the nonsupplemented group. The overall prevalence of preeclampsia was 6.5%, and the lack of a significant intergroup difference might have been due to the small sample size [14]. In a three-arm, double-blind RCT performed in Pakistan, Nausheen et al. followed 350 pregnant women from the first trimester of pregnancy until delivery. The three groups received 4000 IU per day, 2000 IU per day, and 400 IU per day. The intergroup difference in the incidence of preeclampsia was not statistically significant (*p* = 0.99) [30]. There are several possible explanations for this lack of a difference. First, the supplementation in all three arms might have masked subtle differences in the expected outcomes. Second, a possible role of dietary vitamin D in countries with high deficiency rates could not be ruled out. Third, the sample size was small. Last, the absence of a control (nonsupplemented) group might have been due to ethical considerations in a population with known vitamin D deficiency.

Mirzakhani et al.'s study [31] included 816 pregnant women of all parities aged 18–39 years who had been pregnant for between 10 and 18 weeks. The daily dose of cholecalciferol given until delivery was 4400 IU in the intervention arm and 400 IU in the control arm. Overall, the intergroup difference in the frequency of preeclampsia was not statistically significant (8.08% in the intervention arm vs. 8.33% in the control arm: RR [95% CI] = 0.97 [0.61–1.53]). However, Mirzakhani et al.'s subgroup analysis showed that low vitamin D levels in early pregnancy and throughout pregnancy (regardless of the group allocation) were associated with a significantly higher risk of preeclampsia relative to sufficient levels (11.92% vs. 2.25%, respectively; RR [95% CI] = 0.20 [0.06–0.66]; *p* < 0.008). The involvement of vitamin D status in early pregnancy in the development of preeclampsia was inferred from genetic testing, which evidenced the expression of particular genes in the blood of women who developed PE.

In Iran, Karamali et al. conducted a double-blind, placebo-controlled RCT of 60 primigravidae, 30 of whom were considered to be at risk of preeclampsia (i.e., an abnormal uterine artery Doppler wave at 18–20 weeks gestation, a mean resistance index > 0.67 or a pulsatility index > 1.65 and (in some cases) unilateral or bilateral diastolic notches). Pregnant women in the intervention group received 50,000 IU vitamin D twice a month, whereas those in the control group received a placebo multivitamin mineral capsule containing 400 IU vitamin D beginning in the second half of pregnancy [32]. The intergroup difference in the frequency of preeclampsia was not significant (3.3% in the intervention group vs. 10.0% in the control group; *p* = 0.30) [32]. In a double-blind RCT conducted in the USA, Parrish et al. randomised 267 pregnant women (of varying parity) at a 1:1 ratio to an intervention arm (two capsules of a vitamin D-containing phytonutritional supplement) and a control arm. The supplement was taken from 12 WG until delivery [33]. Again, the intergroup difference in the frequency of preeclampsia was not significant (15.9% in the intervention group vs. 16.3% in the control group; RR [95% CI] = 0.97 [0.56–1.69]) [33].

It should be noted that the participants in Karamali et al.'s study were all vitamin D deficient and had very low dietary vitamin D intake at inclusion [32]. A putative difference in pregnancy outcomes in the cohort might have been masked by the administration of vitamin D to both groups (albeit at different doses) or the dose-independent effects of individual factors (e.g., dosing regimens, administration routes, 25(OH)D assays, demographic variables, endogenous vitamin D production, and vitamin D absorption, distribution and excretion). There is still no consensus on how to achieve the optimal (most healthy) blood concentration of 25(OH)D for the skeletal and other organ systems [34].

Parrish et al.'s results [33] can be viewed against the findings of a systematic review of studies of vitamin D_2_ and D_3_ supplementation. The review showed that cholecalciferol produced much greater increases in the total circulating 25(OH) D concentration than ergocalciferol [34, 35]. Furthermore, vitamin D binding protein has less affinity for vitamin D_2_ metabolites than for vitamin D3, which probably explains why the half-life of 25(OH) D_2_ is approximately 10% shorter than that of 25(OH)D_3_ [36]. It is possible that the fortnightly administration in Parrish et al.'s study was intended to circumvent the latter difference. High-dose ergocalciferol intake reduces the 25(OH)D_3_ concentration, probably through competition for 25-hydroxylase [35].

In contrast to studies [30, 32, 37–39] reporting higher vitamin D levels at the end of the trial in supplemented groups than in nonsupplemented groups, we believe that vitamin D should be administered until delivery. Furthermore, it is known that the response to a given amount of vitamin D supplementation (i.e., the increase in the serum 25(OH)D level) depends on the baseline 25(OH)D level: the change is greatest in vitamin-D-deficient people [34]. Furthermore, the amount of vitamin D required to reach a target serum concentration (e.g., 20 ng/mL) depends on several factors, including age, body weight, genetic polymorphisms, and the assay method used [40]. This is consistent with the greater increases observed among the initially vitamin-D-deficient participants in the supplemented group (Fig. 2). It has been established that for a person who is able to absorb vitamin D, supplementation with 100 IU per day results in a serum gain of approximately 0.7 to 1 ng/mL. This increase in serum gain is greater in people with low initial levels and is not linearly proportional to increasing cholecalciferol doses; the serum gain is lower when 25(OH) D levels are higher than 40 ng/mL. Magnesium deficiency can also impair the production of vitamin D, since this metal is an important cofactor in the production of vitamin D and 25(OH)D; magnesium is involved in the binding of cholecalciferol and ergocalciferol to hydroxylation enzymes and vitamin D binding protein [34, 40].

Our results were observed at 34 weeks of pregnancy, i.e., just before the last dose of vitamin D was administered. Although 73.4% of the cases of preeclampsia occurred in the nonsupplemented group, the serum vitamin D levels were higher in the nonsupplemented group (41.47 ± 20.3 ng/mL) than in the supplemented group (37.25 ± 9.4 ng/mL). It is very likely that there were fluctuations in vitamin D levels between the two sampling times; during this time interval, the pregnant women in the nonsupplemented group might have experienced significant vitamin D deficiency and thus had a greater risk of preeclampsia. However, the changes in the proportion of vitamin-D-deficient women in the supplemented and nonsupplemented groups at the end of the trial might confirm the putative effect of vitamin D. Early administration of vitamin D certainly has an impact on reducing the occurrence of preeclampsia, although its effectiveness might also be related to the dose of vitamin D administered and the time point at which supplementation is initiated; these variables should not be ignored when planning preventive interventions. Hence, maintaining target circulating vitamin D levels throughout pregnancy is a key challenge.

Preeclampsia is typically complicated by premature delivery, growth retardation, and low birth weight. A maternal-foetal rescue caesarean section is often performed to avoid the negative outcomes of a prolonged pregnancy. Nausheen et al. did not observe a significant intergroup difference in the frequency of low birth weight (*p* = 0.609) or preterm delivery (*p* = 0.284) [30]. Similarly, Karamali et al. found that in addition to the rates of low birth weight (*p* = 0.15) and preterm delivery (*p* = 0.31), there were no significant intergroup differences in the caesarean section rate (*p* = 0.78), gestational age at delivery (*p* = 0.31), newborn size at birth (*p* = 0.29), and Apgar scores at one and five minutes (*p* = 0.30) [32].

However, some recent cohort studies have shown that birth weight is higher for newborns whose mothers have taken vitamin D supplements during pregnancy. For example, Singh et al. [39] performed a placebo-free RCT in 100 primigravidae (12–16 WG), 50 of whom received 2000 IU vitamin D daily until delivery. The preterm delivery rate was significantly lower in the supplementation group (12.0%) than in the control group (30.0%; *p* = 0.01), and the same was true for the caesarean section rate (8.0% in the supplementation group vs. 38.0% in the control group; *p* = 0.001). For the Apgar score, in the supplementation group, the mean ± SD was higher at one minute (8.38 ± 1.23, vs. 7.10 ± 0.73 in the control group) and five minutes (9.44 ± 1.10, vs. 8.58 ± 1.75, respectively; *p* < 0.05) [39]. Similar conclusions were reached by Sablok et al. [37] after their RCT of 180 primigravidae in India, including 120 women receiving supplementation from 14–20 WG until delivery. The baseline serum vitamin D level was assayed and classified prior to allocation to one of the three study groups (adequate baseline level = a single dose of 60,000 IU vitamin D at 20 WG; inadequate level = two 120,000 IU doses at 20 and 24 WG; deficiency = four 120,000 IU doses at 20, 24, 28 and 32 WG).

The prevalence of preterm delivery was 8.3% in the supplemented group and 21.1% in the nonsupplemented group (*p* = 0.02), whereas the proportion of newborns with a 5-min Apgar score below 7 was higher in the nonsupplemented group (13%, vs. 1.1% in the supplemented group; *p* < 0.001) [37]. In the cohort study performed by Naghshineh et al., the incidence of preterm delivery was 6% in the supplemented group and 24% in the nonsupplemented group (*p* = 0.0056), and the incidence of caesarean section was 49% and 51% (*p* = 0.09), respectively [14]. In the cohort studied by Sasan et al., however, the intergroup difference in the delivery mode was not statistically significant (*p* = 0.88) [29].

For birth weight, the means ± SDs in the supplemented and control groups were 3027 ± 645.7 g vs. 2796 ± 625.2 g (*p* = 0.032) in Naghshineh et al.'s study [(14)], 2600 ± 0.41 g vs. 2400 ± 0.31 g (*p* = 0.01) in Sablok et al.'s study [37], and 3160 ± 0.58 g vs. 2330 ± 0.52 g in Singh et al.'s study [39]. In Sablok et al.'s study, the incidence of low birth weight was significantly higher in the nonsupplemented group (19.2%) than in the supplemented group (8%; *p* < 0.001) [37].

In contrast to some of the above-cited studies, our results suggest that vitamin D supplementation promotes good foetal growth and favourable maternal-foetal outcomes; this might be due to the physiological effects of vitamin D on gestation (greater angiogenesis, effects on renin gene expression, and immunomodulation of inflammatory reactions), the early administration of a suitable dose, the maintenance of an acceptable circulating vitamin D concentration during critical periods of pregnancy, and the decision to provide supplementation in one arm only.

In addition to the comparison of RCT results, robust meta-analyses have provided encouraging conclusions about the value of vitamin D. First, Fogaci et al. found that vitamin D administration during pregnancy was associated with a reduced risk of preeclampsia (OR [95% CI] = 0.37 [0.26–0.52]) and that the risk was even lower when administration was initiated at approximately 20 WG (OR [95% CI] = 0.35 [0.24–0.50]; *p* < 0.001) [41]. Second, the meta-analysis by Andrea Maugeri et al. confirmed the beneficial association among vitamin D supplementation, maternal health, and some neonatal variables (including weight, head circumference, and a reduction in the incidence of low birth weight) [42]. Last, Palacios et al. found that supplementation with vitamin D alone during pregnancy is likely to reduce the risk of preeclampsia (RR = 0.48; 95% CI [0.30–0.79]) and the risk of low birth weight (RR = 0.55; 95% CI [0.35–0.87]). However, Palacios et al. found that vitamin D supplementation was not significantly associated with a lower risk of preterm birth (RR = 0.66; 95% CI [0.34–1.30]). No cases of hypercalcaemia were reported following supplementation [43].

### Strengths and study limitations

First, practical difficulties prevented us from objectively evaluating environmental aspects, such as time spent in the sun, clothing fashion, eating habits, and the level of physical activity. Second, the study did not have double-blind design, i.e., the design usually recommended for this type of investigation. However, this limitation was unlikely to have affected our results because the outcomes were objective variables, the interpretation of which could not be biased. The large and homogeneous sample is a strength of this study. Third, we did not assess the impact on other ethnic groups that did not present to the care facilities. Fourth, a lack of facilities prevented us from exploring genetic aspects of the response to vitamin D supplementation. Difficulties in implementing a large-scale clinical trial are inherent in the Democratic Republic of Congo, and rumours and loss of motivation led to a high drop-out rate. The high drop-out rate was also related to (i) population movements after the eruption of the Nyiragongo volcano in Goma in May 2021 and (ii) the automatic deduction of the “*Registre des Appareils Mobiles”* tax on phone credit (prompting the loss of telephone contacts and preventing us from collecting foetal annex data at the delivery sites).

## Conclusion

The present results argue in favour of vitamin D supplementation in pregnant woman and clearly suggest that the risks of preeclampsia, preterm delivery and caesarean delivery are lower when the serum vitamin D level is maintained in the normal range throughout pregnancy. The high live birth rate, high in utero weight and birth size provide support in favour of vitamin D administration. The timing of vitamin D administration and the achievement of an optimal serum concentration are likely to be important factors. These observations encourage the preventive administration of vitamin D in sufficient dose during preconception consultations or antenatal care to hope to obtain, in a given context, a reduction in maternal-foetal morbidity and mortality secondary to PE.

## Data Availability

The data used during the study are available from the corresponding author upon reasonable request.

## Abbreviations

IU: International unit
25(OH)D: 25 Hydroxyvitamin D
RR: Relative risk
PE: Preeclampsia
SBP: Systolic blood pressure
DBP: Diastolic blood pressure
GW: Gestational week
GA: Gestational age
CI: Confidence interval
CRD: Congo red Dot Test
P: Probability
RCT: Randomised controlled trial
Cm: Centimetre
Min: Minute
VEGF: Vascular endothelial growth factor

## References

[1] Lo JO, Mission JF, Caughey AB (2013). Hypertensive disease of pregnancy and maternal mortality. Curr Opin Obstet Gynecol.

[2] Abalos E, Cuesta C, Grosso AL, Chou D, Say L (2013). Global and regional estimates of preeclampsia and eclampsia: a systematic review. Eur J Obstet Gynecol Reprod Biol.

[3] Duley L (2003). Pre-eclampsia and the hypertensive disorders of pregnancy. Br Med Bull.

[4] O’Callaghan KM, Kiely M (2018). Systematic review of vitamin D and hypertensive disorders of pregnancy. Nutrients.

[5] Vata PK, Chauhan NM, Nallathambi A, Hussein F (2015). Assessment of prevalence of preeclampsia from Dilla region of Ethiopia. BMC Res Notes.

[6] Tsatsaris V, Fournier T, Winer N (2008). Physiopathologie de la prééclampsie. J Gynecol Obstet Biol Reprod.

[7] Kabuseba RK, Tugirimana PL, Moyene JPE, Kalume XK, Zambèze JBKS (2022). Calcemia, Vitamin D and seasonal influences in preeclampsia in Goma. GSC Adv Res Rev.

[8] Preeclampsia: Pathogenesis - UpToDate. [cité 11 août 2023]. Disponible sur: https://www.uptodate.com/contents/preeclampsia-pathogenesis?search=Preeclampsia%20MD.%20Pathogenesis.%20Uptodate;%202018.%20&source=search_result&selectedTitle=1~150&usage_type=default&display_rank=1

[9] Laganà AS, Giordano D, Loddo S, Zoccali G, Vitale SG, Santamaria A (2017). Decreased Endothelial Progenitor Cells (EPCs) and increased Natural Killer (NK) cells in peripheral blood as possible early markers of preeclampsia: a case-control analysis. Arch Gynecol Obstet.

[10] Petca A, Sinescu R, Sandru F, Petca RC, Dumitrascu M, Mehedintu C (2022). New approaches in predicting and diagnosing preeclampsia: Congo Red Dot Paper Test (Review). Exp Ther Med.

[11] Nema J, Sundrani D, Joshi S (2019). Role of vitamin D in influencing angiogenesis in preeclampsia. Hypertens Pregnancy.

[12] Benachi A, Baptiste A, Taieb J, Tsatsaris V, Guibourdenche J, Senat MV (2020). Relationship between vitamin D status in pregnancy and the risk for preeclampsia: A nested case-control study. Clin Nutr.

[13] Richard KK, Marcelline BS, Jean-Pierre EM, Pierrot LT, Prosper KMK, Jean-Baptiste KSZ (2020). Vitamin D Status and the Determinants of Preeclampsia in Pregnant Women in Goma (Democratic Republic of the Congo). Open J Obst Gynecol.

[14] Naghshineh E, Sheikhaliyan S. Effect of vitamin D supplementation in the reduce risk of preeclampsia in nulliparous women. Advanced Biomedical Research. 2016;5. 10.4103/2277-9175.175239 PMC4770602 26962509

[15] Robinson CJ, Alanis MC, Wagner CL, Hollis BW, Johnson DD (2010). Plasma 25-hydroxyvitamin D levels in early-onset severe preeclampsia. Am J Obstet Gynecol.

[16] Hilger J, Friedel A, Herr R, Rausch T, Roos F, Wahl DA (2014). A systematic review of vitamin D status in populations worldwide. Br J Nutr.

[17] Bodnar LM, Catov JM, Simhan HN, Holick MF, Powers RW, Roberts JM (2007). Maternal vitamin D deficiency increases the risk of preeclampsia. J Clin Endocrinol Metab.

[18] mondiale de la Santé O. Recommendations de l’OMS pour la prévention et le traitement de la prééclampsie et de l’éclampsie. 2014;

[19] Benachi A, Cordier AG, Courbebaisse M, Souberbielle JC (2013). Vitamine D et grossesse. La Presse Médicale.

[20] Hollis BW, Wagner CL (2013). Vitamin D and pregnancy: skeletal effects, nonskeletal effects, and birth outcomes. Calcif Tissue Int févr.

[21] Hollis BW, Johnson D, Hulsey TC, Ebeling M, Wagner CL (2011). Vitamin D supplementation during pregnancy: double-blind, randomized clinical trial of safety and effectiveness. J Bone Miner Res.

[22] Hollis BW (2019). Vitamin D status during pregnancy: The importance of getting it right. EBioMedicine.

[23] Rostami M, Tehrani FR, Simbar M, BidhendiYarandi R, Minooee S, Hollis BW (2018). Effectiveness of Prenatal Vitamin D Deficiency Screening and Treatment Program: A Stratified Randomized Field Trial. J Clin Endocrinol Metab.

[24] Haugen M, Brantsæter AL, Trogstad L, Alexander J, Roth C, Magnus P (2009). Vitamin D supplementation and reduced risk of preeclampsia in nulliparous women. Epidemiology.

[25] De-Regil LM, Palacios C, Lombardo LK, Peña-Rosas JP. Vitamin D supplementation for women during pregnancy. Cochrane database of systematic reviews. 2016;(1). 10.1002/14651858.CD008873.pub3 26765344

[26] Elongi JP, Tandu B, Spitz B, Verdonck F (2011). Influence de la variation saisonnière sur la prévalence de la pré-éclampsie à Kinshasa. Gynécologie Obstétrique Fertilité.

[27] Program NHBPE. Report of the national high blood pressure education program working group on high blood pressure in pregnancy Am J Obstet Gynecol 2000;183(1):s1‑22. 10920346

[28] Torchin H, Ancel PY (2016). Epidemiology and risk factors of preterm birth. J Gynecol Obstet Biol Reprod.

[29] Behjat Sasan S, Zandvakili F, Soufizadeh N, Baybordi E. The effects of vitamin D supplement on prevention of recurrence of preeclampsia in pregnant women with a history of preeclampsia. Obstetrics and gynecology international. 2017;2017. 10.1155/2017/8249264 PMC5585545 28912817

[30] Nausheen S, Habib A, Bhura M, Rizvi A, Shaheen F, Begum K (2021). Impact evaluation of the efficacy of different doses of vitamin D supplementation during pregnancy on pregnancy and birth outcomes: a randomised, controlled, dose comparison trial in Pakistan. BMJ Nutr Prev Health.

[31] Mirzakhani H, Litonjua AA, McElrath TF, O’Connor G, Lee-Parritz A, Iverson R (2016). Early pregnancy vitamin D status and risk of preeclampsia. J Clin Investig.

[32] Karamali M, Beihaghi E, Mohammadi AA, Asemi Z (2015). Effects of high-dose vitamin D supplementation on metabolic status and pregnancy outcomes in pregnant women at risk for pre-eclampsia. Horm Metab Res.

[33] Parrish MR, Martin JN, Lamarca BB, Ellis B, Parrish SA, Owens MY (2013). Randomized, placebo controlled, double blind trial evaluating early pregnancy phytonutrient supplementation in the prevention of preeclampsia. J Perinatol.

[34] Dominguez LJ, Farruggia M, Veronese N, Barbagallo M (2021). Vitamin D sources, metabolism, and deficiency: available compounds and guidelines for its treatment. Metabolites.

[35] Sosa Henríquez M, Gómez de Tejada Romero MJ (2020). Cholecalciferol or calcifediol in the management of vitamin D deficiency. Nutrients.

[36] Bouillon R, Verlinden L, Verstuyf A. Is vitamin D2 really bioequivalent to vitamin D3? Oxford University Press; 2016. 10.1210/en.2016-1528 27580803

[37] Sablok A, Batra A, Thariani K, Batra A, Bharti R, Aggarwal AR (2015). Supplementation of vitamin D in pregnancy and its correlation with feto-maternal outcome. Clin Endocrinol.

[38] Roth DE, Al Mahmud A, Raqib R, Akhtar E, Perumal N, Pezzack B (2013). Randomized placebo-controlled trial of high-dose prenatal third-trimester vitamin D3 supplementation in Bangladesh: the AViDD trial. Nutr J.

[39] Singh J, Hariharan C, Bhaumik D (2015). Role of vitamin D in reducing the risk of preterm labour. Int J Reprod Contracept Obstet Gynecol.

[40] Bilezikian JP, Formenti AM, Adler RA, Binkley N, Bouillon R, Lazaretti-Castro M (2021). Vitamin D: Dosing, levels, form, and route of administration: Does one approach fit all?. Rev Endocr Metab Disord déc.

[41] Fogacci S, Fogacci F, Banach M, Michos ED, Hernandez AV, Lip GY (2020). Vitamin D supplementation and incident preeclampsia: a systematic review and meta-analysis of randomized clinical trials. Clin Nutr.

[42] Maugeri A, Barchitta M, Blanco I, Agodi A (2019). Effects of vitamin D supplementation during pregnancy on birth size: a systematic review and meta-analysis of randomized controlled trials. Nutrients.

[43] Palacios C, Kostiuk LK, Peña-Rosas JP. Vitamin D supplementation for women during pregnancy. Cochrane Database of Systematic Reviews. 2019;(7). 10.1002/14651858.CD008873.pub4 PMC6659840 31348529

